# Long noncoding RNA MEG3 suppresses liver cancer cells growth through inhibiting β-catenin by activating PKM2 and inactivating PTEN

**DOI:** 10.1038/s41419-018-0305-7

**Published:** 2018-02-15

**Authors:** Qidi Zheng, Zhuojia Lin, Jie Xu, Yanan Lu, Qiuyu Meng, Chen Wang, Yuxin Yang, Xiaoru Xin, Xiaonan Li, Hu Pu, Xin Gui, Tianming Li, Wujun Xiong, Dongdong Lu

**Affiliations:** 1 Research Center for Translational Medicine at Shanghai East Hospital, School of Life Science and Technology, Tongji University, 200092 Shanghai, China; 2 Department of Hepatology, Shanghai East Hospital, Tongji University School of Medicine, 200120 Shanghai, China

## Abstract

Maternally expressed gene 3 (MEG3) encodes an lncRNA which is suggested to function as a tumor suppressor and has been showed to involve in a variety of cancers. Herein, our findings demonstrate that MEG3 inhibits the malignant progression of liver cancer cells in vitro and in vivo. Mechanistically, MEG3 promotes the expression and maturition of miR122 which targets PKM2. Therefore, MEG3 decreases the expression and nuclear location of PKM2 dependent on miR122. Furthermore, MEG3 also inhibits CyclinD1 and C-Myc via PKM2 in liver cancer cells. On the other hand, MEG3 promotes β-catenin degradation through ubiquitin–proteasome system dependent on PTEN. Strikingly, MEG3 inhibits β-catenin activity through PKM2 reduction and PTEN increase. Significantly, we also found that excessive β-catenin abrogated the effect of MEG3 in liver cancer. In conclusion, our study for the first time demonstrates that MEG3 acts as a tumor suppressor by negatively regulating the activity of the PKM2 and β-catenin signaling pathway in hepatocarcinogenesis and could provide potential therapeutic targets for the treatment of liver cancer.

## Introduction

Recent research has found that long noncoding RNAs (lncRNAs) were involved in various human cancers. Maternally expressed gene 3 (MEG3) has been shown to be involved in a variety of cancers and is downregulated in most cancers and affects cell proliferation, progression, and prognosis^1–5^. Notably, genetic variants and imprint change in MEG3 may contribute to the development and risk of cancer^6,7^. Moreover, MEG3 increases autophagy^8^, and epigenetic repression of MEG3 represses the p53 pathway and enhances Wnt/β-catenin signaling^9,10^. In addition, MEG3 produces an antitumor effect in several cancers^11,12^. Furthermore, MEG3 functions as a competing endogenous RNA to regulate cancer progression^13^ and TGF-β pathway genes through the formation of RNA–DNA triplex structures^14^. Strikingly, excessive MEG3 promotes osteogenic differentiation of mesenchymal stem cells from multiple myeloma patients by targeting BMP4 transcription^15^.

miR-122 is involved in human cancer proliferation, invasion, and progression^16–19^. In particular, miR-122 reverses the drug resistance and hepatotoxicity in hepatocellular carcinoma cells through regulating the tumor metabolism^20,21^. Pyruvate kinase muscle isozyme M2 (PKM2) is a limiting glycolytic enzyme that catalyzes the final step in glycolysis, which is key in tumor metabolism and growth^22,23^. Moreover, PKM2 plays a pivotal role in the growth, survival, and metabolic reprogramming of cancer cells^24,25^. Notably, loss of SIRT2 function in cancer cells reprograms their glycolytic metabolism via PKM2 regulation^26^. In addition, our previous study indicates that double mutant P53 (N340Q/L344R) promotes hepatocarcinogenesis mediated by PKM2^27^. Phosphatase and tensin homolog (PTEN) is one of the powerful switches for the conversion between tumor suppressors and oncogenes. A number of studies have suggested that PTEN may alter various functions of certain oncogenic proteins^28–33^. Strikingly, PTEN opposes malignant transformation of pre-B cells and breast cells^34,35^. In particular, the PI3K-PTEN-AKT-mTOR pathway is a central controller of cell growth and a key driver for human cancer^36^. β-catenin (encoded by CTNNB1) is a subunit of the cell surface cadherin protein complex that acts as an intracellular signal transducer in the WNT signaling pathway. Many hepatic tumors such as hepatocellular adenomas, hepatocellular cancers, and hepatoblastomas have mutations in β-catenin that result in constitutive activation of β-catenin^37^. Also, Wnt/β-catenin/TCF-4 signaling is crucial for the proliferation and self-renewal maintenance of cancer stem cells^38–41^. Strikingly, MSK1-mediated β-catenin phosphorylation confers resistance to PI3K/mTOR inhibitors in glioblastoma^42^.

In the present study, we indicate that MEG3 inhibits the malignant progression of liver cancer cells in vitro and in vivo. Our study for the first time demonstrated that MEG3 acts as a tumor suppressor by negatively regulating the activity of the PKM2 and β-catenin pathway in hepatocarcinogenesis and may provide potential therapeutic targets for the treatment of liver cancer.

## Experimental material and procedures

### Cell lines and plasmids

Human liver cancer line Hep3B was maintained in DMEM medium supplemented with 10% heat-inactivated fetal bovine serum (FBS) (Gibco) in a humidified atmosphere of 5% CO_2_ incubator at 37 °C. Plasmids pGFP-V-RS, pCMV6-A-GFP, pCMV6-XL5-β-catenin, pCMV6-XL5-PTEN, pGFP-V-RS-PTEN, pGFP-V-RS-β-catenin, and pMiR-Target were purchased from Origene (Rockville, MD 20850, USA). pEGP-miR122(BioLab), pCMV6-A-GFP–MEG3 was constructed in our lab.

### Cell transfection and stable cell lines

Cells were transfected with DNA plasmids using transfast transfection reagent lipofectamine^R^ 2000 (Invitrogen) according to manufacturer’s instructions. For screening stable cell lines, 48 h after transfection, the cells were plated in the selective medium containing G418 (1000–2000 μg/ml, Invitrogen) or Puromycin (1–2 μg/ml, Calbiochem) for about 4 weeks or so, and the GFP-positive cells were selected and the selective media were replaced every 3 days.

### RT-PCR

Total RNA was purified using Trizol (Invitrogen) according to manufacturer’s instructions. cDNA was prepared by using oligonucleotide (dT)_17-18_, random primers, and a SuperScript First-Strand Synthesis System (Invitrogen). The PCR reaction was performed in 36 cycles with each cycle consisting of a denaturation step (94 °C for 30 s, and 3 min for the first cycle only), an annealing step (56 °C (MEG3) or 55 °C (PKM2 and β-actin) or 58 °C (pre-miR122) for 30 s), and an elongation step (72 °C for 30 s, 10 min for the last cycle only). PCR primers: MEG3:P1: 5′-TCCATGCTGAGCTGCTGCCAAG-3′; P2: 5′- AGTCGACAAAGACTGACACCC-3′; PKM2P1: 5′-GCCACCATGTCGAAGCCCCATA-3′; P2: 5′-TCACGGCACAGGAACAACACGC-3′; pre-miR122: P1: 5′-TTGCCTAGCAGTAGCTATTT-3′; P2: 5′GGCTACAGAGTTTCCTTAGC-3′. β-actin was used as an internal control.

### MicroRNA detection

Total RNA was isolated from cultured cells using Trizol (Invitrogen, Carlsbad, CA, USA) according to the manufacturer’s protocol. Real-time RT-PCR-based detection of mature miR-122 and U6 snRNA was achieved with the miRNA Detection kit (including a universal primer, U6 primers, Qiagen) and miR122 specific upstream primers (mature miR122:P1: 5′-TGGAGTGTGACAATGGTGTTTG-3′ Origene, USA). qRT-PCR was performed with a StepOne Plus real-time PCR system (Applied Biosystems). The real-time PCR reaction was performed in 40 cycles with each cycle consisting of a denaturation step (95 °C for 15 s, and 15 min for the first cycle only) and an annealing step (60 °C for 30 s). Each sample was run in triplicate. *C*_t_ values for miR122 were calculated and normalized to *C*_t_ values for U6 snRNA.

### Western blotting

The logarithmically growing cells were washed twice with ice-cold phosphate-buffered saline (PBS, Hyclone) and lysed in a RIPA lysis buffer. Cells lysates were centrifuged at 12,000*g* for 20 min at 4 °C after sonication on ice, and the supernatant was separated. After being boiled for 5–10 min in the presence of 2-mercaptoethanol, the samples containing cellulars proteins were separated on a 10% sodium dodecyl sulfate-polyacrylamide gel electrophoresis (SDS-PAGE) and transferred onto a nitrocellulose membrane, blocked in 10% dry milk-TBST (20 mM Tris-HCl (pH 7.6), 127 mM NaCl, 0.1% Tween 20) for 1 h at 37 °C. Following three washes in Tris-HCl pH 7.5 with 0.1% Tween 20, the blots were incubated with 0.2 µg/ml of antibody (appropriate dilution) overnight at 4 °C. Following three washes, the membranes were then incubated with secondary antibody for 60 min at 37 °C or 4 °C overnight in TBST. Signals were visualized by ODYSSEY infrared imaging system (LI-COR, Lincoln, Nebraska, USA). IRDye 680LT/IRDye 800CW secondary antibodies (1:50,000) were purchased from LI-COR Scientific Company. Standard Western blotting procedures were used with the following antibodies: anti-PCNA (Santa Cruz, Biotech), anti-CTCF (Santa Cruz, Biotech), anti-CREB (Santa Cruz, Biotech), anti-RNA polII(Abcam), anti-H3K9me3 (Abcam), anti-H3K36me3 (Abcam), anti-PKM2 (Abcam), anti-ERK1/2 (Abcam), anti-pPKM2(ser37) (Cell signaling), anti-Histone H3 (Abcam), anti-H3K9Ac (Abcam), anti-METTL3 (Santa Cruz, Biotech), anti-AKT (Abcam), anti-pAKT (Abcam), anti-GSK3β(ser9), anti-GSK3β(Tyr216), anti-GSK3β (Santa Cruz, Biotech), anti-β-catenin (Santa Cruz, Biotech), anti-HA, anti-Skp2 (Santa Cruz, Biotech), anti-TCF4 (Santa Cruz, Biotech), anti-LEF (Santa Cruz, Biotech), anti-PTEN (Santa Cruz, Biotech), anti-LEF (Santa Cruz, Biotech), anti-TCF4 (Santa Cruz, Biotech), anti-C-myc (Santa Cruz, Biotech), and anti-cyclinD1 (Santa Cruz, Biotech).

### Co-immunoprecipitation (IP)

Cells were lysed in 1 ml of whole-cell extract buffer A (50 mM pH 7.6 Tris-HCl, 150 mM NaCl, 1% NP40, 0.1 mM EDTA, 1.0 mM DTT, 0.2 mM PMSF, 0.1 mM Pepstatine, 0.1 mM Leupeptine, 0.1 mM Aproine); 500 μl cell lysates were used for IP with antibody. In brief, protein was pre-cleared with 30 μl protein G/A-plus agarose beads (Santa Cruz, Biotechnology, Inc., CA) for 1 h at 4 °C and the supernatant was obtained after centrifugation (5000 rpm) at 4 °C. Pre-cleared homogenates (supernatant) were incubated with 2 µg of antibody and/or normal mouse/rabbit IgG with rotation for 4 h at 4 °C. The immunoprecipitates were incubated with 30 μl protein G/A-plus agarose beads by rotation overnight at 4 °C, and then centrifuged at 5000 rpm for 5 min at 4 °C. The precipitates were washed five times for 10 min with beads wash solution (50 mM pH 7.6 Tris-HCl, 150 mM NaCl, 0.1% NP-40, 1 mM EDTA), resuspended in 60 µl 2 × SDS-PAGE sample loading buffer, and incubated for 5–10 min at 100 °C. Western blotting was performed with related antibodies.

### RNA immunoprecipitation (RIP)

Cells were lysed (15 min, 0 °C) in 100 mM KCl, 5 mM MgCl_2_, 10 mM HEPES [pH 7.0], 0.5% NP40, 1 mM DTT, 100 units/ml RNase OUT (Invitrogen), 400 μM vanadyl–ribonucleoside complex, and protease inhibitors (Roche), clarified and stored at −80 °C. Ribonucleoprotein particle-enriched lysates were incubated with protein A/G-plus agarose beads (Santa Cruz, Biotechnology, Inc., CA) together with the antibody or normal mouse or rabbit IgG for 4 h at 4 °C. Beads were subsequently washed four times with 50 mM Tris-HCl (pH 7.0), 150 mM NaCl, 1 mM MgCl_2_, and 0.05% NP-40, and twice after addition of 1 M urea. Immunoprecipitates were digested with proteinase K (55 °C; 30′) and mRNAs were isolated and purified, and then RT-PCR was performed.

### Super-EMSA (gel-shift)

Cells were washed and scraped in ice-cold PBS to prepare nuclei for electrophoretic gel mobility shift assay with the use of the gel shift assay system modified according to the manufacturer’s instructions (Promega). In brief, consensus oligonucleotides for damage or repair DNA were biotin-labeled (hot probe). Each binding reaction was carried out with 1 µg biotinylated dsDNA probe and 200 µg purified nuclear protein in 20 µl of binding buffer containing 0.5 mg/ml poly(dI:dC) (25 mM HEPES at pH 8.0 with 50 mM KCl, 0.1% Triton X100, 2 mM MgCl_2_, 3 mM DTT, and 5% glycerol). Twenty-five pmol unlabeled cold DNA motifs (a 250-fold excess) were added in the competition assays. Reactions were carried out for 30 min incubation at room temperature, followed by overnight incubation at 4 °C. Reaction mixtures were loaded onto 6% TBE polyacrylamide gels and separated in 0.5%×TBE at 100 v on ice until the dye front migrated two-thirds of the way to NC membranes and Western blotting was performed for anti-biotin.

### Dual luciferase reporter assay

Cells were transfected with luciferase construct plasmids and pRL-tk. After incubation for 48 h, the cells were harvested with Passive Lysis Buffer (Promega), and luciferase activities of cell extracts were measured with the use of the Dual luciferase assay system (Promega) according to manufacturer’s instructions. Luciferase activity was measured and normalized for transfection efficiency with Renilla luciferase activity.

### Chromatin immunoprecipitation (CHIP)

Cells were cross-linked with 1% (v/v) formaldehyde for 10 min at room temperature and stopped with 125 mM glycine for 5 min. Cross-linked cells were washed with PBS, resuspended in lysis buffer, and sonicated for 8–10 min in a SONICS. Chromatin extracts were pre-cleared with Protein-A/G-Sepharose beads, and immunoprecipitated with specific antibody on Protein-A/G-Sepharose beads. After washing, elution, and de-cross-linking, the ChIP DNA was detected by PCR. The following primer pairs were used: C-myc CHIP promoter: P1: 5′-TAACTCGCTGTAGTAATTCC-3′; P2: 5′-CCCTATGGGCAAAGTTTCGT-3′; CyclinD1 CHIP promoter: P1: 5′-GAAGAGTCTCCAGGCTAGAA-3′; P2: 5′-TTGTAGCCTGGAGACTCTTC-3′.

### Cells proliferation CCK8 assay

Cells were synchronized in G0 phase by serum deprivation and then released from growth arrest by re-exposure to serum, and then cells were grown in complete medium for assay. The cell proliferation reagent CCK8 was purchased from Roch and the operation was carried out according to the manufacturer's instruction. In brief, cells at a concentration 4 × 10^3^ were seeded into 96-well culture plates in 100 μl culture medium containing 10% heat-inactivated fetal calf serum (FCS). Before detection, 10 μg/well cell proliferation reagent CCK8 was added and incubated for 4 h at 37 °C and 5% CO_2_. Cell growth curve was based on the corresponding normalized values of OD450 and each point represents the mean of three independent samples.

### Colony-formation efficiency assay

5 × 10^2^ cells were plated on a 10-cm dish, then 10 ml DMEM containing 10% FBS was added into each 10-cm dish of the three replicates. Then these dishes were incubated at 37 °C in a humidified incubator for 10 days. The cell colonies on the dishes were stained with 1 ml of 0.5% Crystal Violet for more than 1 h and the colonies were counted.

### Xenograft transplantation in vivo

Four-weeks-old male athymic Balb/C mice were purchased from Shi Laike Company (Shanghi, China) and maintained in the Tongji animal facilities approved by the China Association for Accreditation of Laboratory Animal Care. The athymic Balb/C mice were injected in the armpit area subcutaneously with Hep3B suspension of 1 × 10^6^ cells in 100 μl of PBS. The mice were observed over 4 weeks, and then sacrificed to recover the tumors. The wet weight of each tumor was determined for each mouse. A portion of each tumor was fixed in 4% paraformaldehyde and embedded in paraffin for histological hematoxylin–eosin (HE) staining.

### Ethics statement

All methods were carried out in “accordance” with the approved guidelines. All experimental protocols “were approved by” a Tongji university institutional committee. Informed consent was obtained from all subjects. The use of mice  was reviewed and approved by the China national institutional animal care and use committee”.

## Results

### MEG3 inhibits liver cancer cell growth in vitro and in vivo

**Fig. 1:**
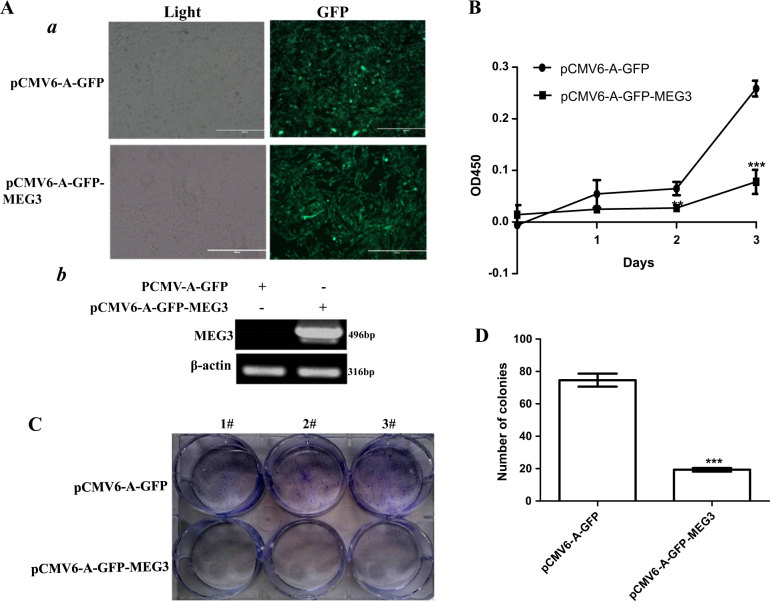
MEG3 inhibits liver cancer cell growth in vitro. **A**a The photography of the Hep3B cell lines transfected with pCMV6-A-GFP or pCMV6-A-GFP-MEG3. b RT-PCR for MEG3 in MEG3 overexpressed control Hep3B stable cell lines; β-actin as an internal control. **B** Cell proliferation assay was performed in 96-well format using the CCK8 cells proliferation kit to determine the cell viability as described by the manufacturer. Each sample was assayed in triplicates for 3 days consecutively. Cell growth curve was based on the corresponding values of OD450 and each point represents the mean of three independent samples. Data are means of values from three independent experiments, mean ± SEM. **, *P* < 0.01;*, *P* < 0.05. **C** The photography of the colonies from the cell lines indicated in left.** D** Cell plate colony formation ability assay. Data are means of values from three independent experiments, mean ± SEM. **, *P* < 0.01;*, *P* < 0.05

**Fig. 2:**
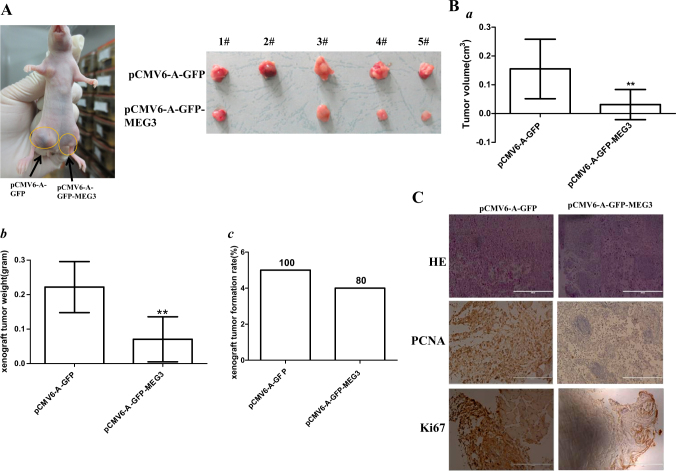
MEG3 inhibits liver cancer cell growth in vivo. **A** The photography of xenograft tumors from Balb/C null mouse injected with Hep3B cells transfected with pCMV6-A-GFP, pCMV6-A-GFP-MEG3 subcutaneously in the armpit. **B** The xenograft tumors weight (gram) in the four groups indicated in left. Data are means of values from nine Balb/c mice, mean ± SEM, *n* = 5, *, *P* < 0.05; **, *P* < 0.01. **C** A portion of each xenograft tumor was fixed in 4% formaldehyde and embedded in paraffin, and micrometers of sections (4 µm) were made for hematoxylin–eosin (HE) staining (original magnification ×100), and anti-PCNA and anti-k67 immunostainning in xenograft tumor samples (original magnification ×100)

To investigate whether MEG3 inhibited the malignant growth of human liver cancer cell line Hep3B, we first established two stable Hep3B cell lines transfectd with pCMV6-A-GFP (GFP ctrl), pCMV6-A-GFP-MEG3 (MEG3), respectively. As shown in Fig. 1a, the expression of MEG3 was significantly increased in MEG3 overexpressing Hep3B on the transcriptional level. As shown in Fig. 1b, excessive MEG3 significantly decreased the growth of liver cancer cell Hep3B compared to the control cells (*P* < 0.01). We further performed plate colony formation assay and observed a significant decrease in colony formation efficiency rate in excessive MEG3 (74.67 ± 4.04 versus 19.33 ± 1.15, *P* = 1.0957E−05 <0.01) (Fig. 1c, d). To explore the effect of MEG3 on liver cancer cells in vivo, the two stable Hep3B were injected subcutaneously into athymic Balb/C mice. As shown in Fig. 2a–c, when MEG3 was overexpressed, the xenograft tumor weight decreased approximately one-third compared to the corresponding control group (0.22152 ± 0.07382 g versus 0.07042 ± 0.0652 g, *P* = 0.004061372 <0.01); when MEG3 was overexpressed, the xenograft tumor size decreased approximately one-fifth compared to the corresponding control group (0.15508 ± 0.1035 cm^3^ versus 0.03125 ± 0.05229 cm^3^, *P* = 0.007228 <0.01). Moreover, compared to control, xenograft tumors contained less of poorly differentiated cells in MEG3 overexpression group (Fig. 2d, upper). The proliferation index (calculated as percentage of PCNA-positive cells) and Ki67 were significantly lower in MEG3 overexpressing xenograft tumors compared to the control group (Fig. 2d, middle and lower). Taken together, these findings demonstrate that MEG3 inhibits malignant progression of liver cancer cells in vitro and in vivo.

### MEG3 promotes the expression and mature of miR122 which targets for PKM2 and inhibits expression of PKM2

**Fig. 3:**
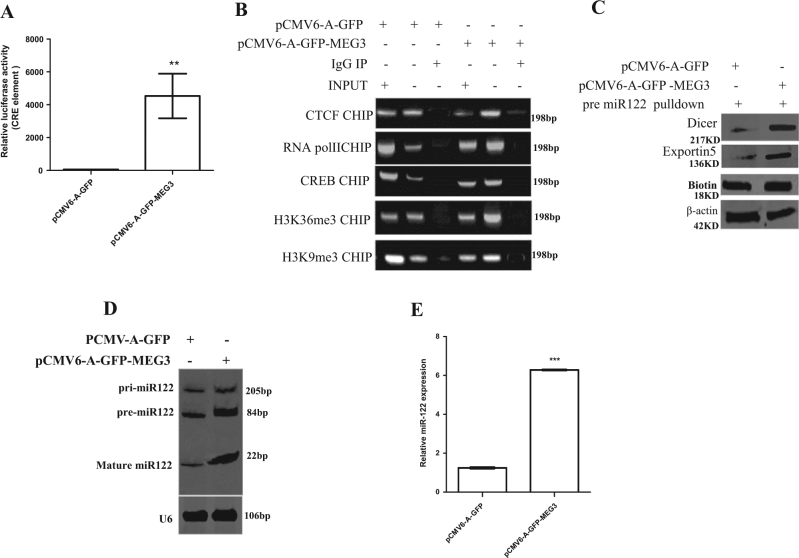
MEG3 inhibits expression of miR122. **A** CRE binding element luciferase activity assay in liver cancer cells Hep3B cell lines transfected with pCMV6-A-GFP, pCMV6-A-GFP-MEG3, respectively. **B** Chromatin immunoprecipitation (CHIP) with anti-CTCF, anti-CREB, anti-RNA polII, anti-H3K9me3, anti-H3K36me3 followed by PCR with pri-miR122 promoter primers in liver cancer cells Hep3B cell lines transfected with pCMV6-A-GFP, pCMV6-A-GFP-MEG3, respectively. IgG CHIP as a negative control; miR122 promoter as INPUT. **C** Biotin-pre-miR122 pulldown followed by Western blotting with anti-Dicer, anti-Exportin5. Biotin as INPUT and β-actin as an internal control. **D** Northern–Western blotting analysis of miR122 in liver cancer cells Hep3B cell lines transfected with pCMV6-A-GFP, pCMV6-A-GFP-MEG3. U6 as an internal control. **E** The real-time PCR detection of mature miR122 in liver cancer cells transfected with pCMV6-A-GFP, pCMV6-A-GFP-MEG3, respectively. Each value was presented as mean ± standard error of the mean (SEM). **, *P* < 0.01

**Fig. 4:**
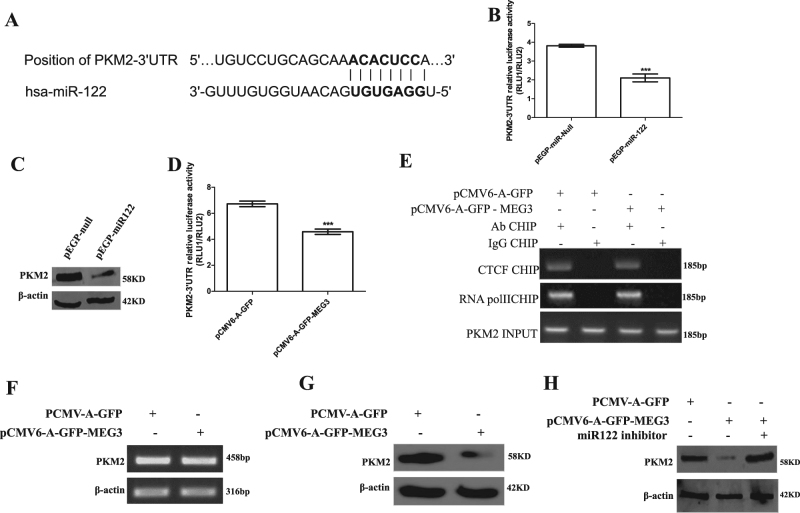
MEG3 inhibits expression of PKM2 via miR122. **A** The bioinformatics analysis of mir122 targeting for PKM2. **B** PKM2 3′-UTR luciferase activity assay in liver cancer cells Hep3B cell lines transfected with pEGP-null, pEGP-miR122, respectively. Data are means of values from three independent experiments, mean ± SEM. **, *P* < 0.01; *, *P* < 0.05. **C** The Western blotting with anti-PKM2 in liver cancer cells Hep3B cell lines transfected with pEGP-null, pEGP-miR122, respectively. β-actin as an internal control. **D** PKM2 3′-UTR luciferase activity assay in liver cancer cells Hep3B cell lines transfected with pCMV6-A-GFP, pCMV6-A-GFP-MEG3, respectively. Data are means of values from three independent experiments, mean ± SEM. **, *P* < 0.01; *, *P* < 0.05. **E** Chromatin immunoprecipitation (CHIP) with anti-CTCF, anti-RNA polII, followed by PCR with PKM2 promoter primers in liver cancer cells Hep3B cell lines transfected with pCMV6-A-GFP, pCMV6-A-GFP-MEG3, respectively. IgG CHIP as negative control. PKM2 promoter as INPUT. **F** RT-PCR analysis with PKM2 primers in liver cancer cells Hep3B cell lines transfected with pCMV6-A-GFP, pCMV6-A-GFP-MEG3, respectively. β-actin as an internal control. **G** Western blotting analysis with anti-PKM2 in liver cancer cells Hep3B cell lines transfected with pCMV6-A-GFP, pCMV6-A-GFP-MEG3, respectively. β-actin as internal control. **h**. Western blotting analysis with anti-PKM2 in liver cancer cells Hep3B cell lines transfected with pCMV6-A-GFP, pCMV6-A-GFP-MEG3, pCMV6-A-GFP-MEG3 plus mi122 inhibitor., respectively. β-actin as an internal control

To address whether MEG3 influences the expression and maturation of miR122, we performed related experiments in MEG3 or miR122 overexpressing Hep3B cell line. As shown in Fig. 3a, MEG3 could enhance the CRE luciferase activity in Hep3B cells. Moreover, MEG3 overexpression enhanced the loading of CTCF, CREB, H3K9me3, H3K36me3, RNA polII on the miR122 promoter region (Fig. 3b). Furthermore, MEG3 overexpression enhanced the binding of Dicer, Exportin5 to the pre-miR122 probe (Fig. 3c). Importantly, the pei-miR122, pre-miR122, and mature miR122 were significantly increased in excessive MEG3 group compared to the control group (Fig. 3d, e). Surprisingly, miR122 targets for 3′-untranslational region (UTR) of PKM2 (Fig. 4a), and inhibits PKM2 3-UTR luciferase activity (Fig. 4b) and PKM2 expression (Fig. 4c). Taken together, MEG3 promotes the expression and maturation of miR122 which targets PKM2 and inhibits the expression of PKM2.

### MEG3 inhibits localization and function of PKM2

**Fig. 5:**
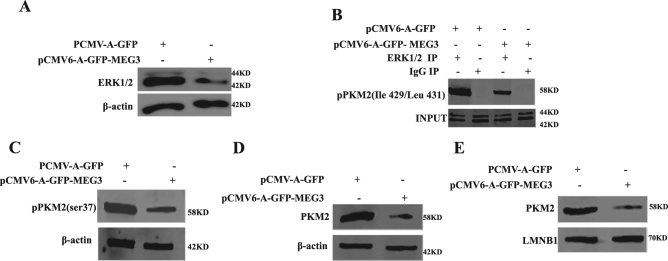
MEG3 inhibits nucear localization of PKM2. **A** Western blotting analysis with anti-ERK1/2 in liver cancer cells Hep3B cell lines transfected with pCMV6-A-GFP, pCMV6-A-GFP-MEG3, respectively. β-actin as an internal control. **B** Co-immunoprecipitation (IP) with anti-ERK1/2 followed by Western blotting with anti-pPKM2(Ile 429/Leu 431) in the Hep3B cell lines transfected with pCMV6-A-GFP, pCMV6-A-GFP-MEG3, respectively. IgG IP as negative control. INPUT refers to Western blotting with anti-pPKM2(Ile 429/Leu 431). **C** Western blotting analysis with anti-pPKM2(ser37) in liver cancer cells Hep3B cell lines transfected with pCMV6-A-GFP, pCMV6-A-GFP-MEG3, respectively. β-actin as an internal control. **D** Western blotting analysis with anti-PKM2 in liver cancer cells Hep3B cell lines transfected with pCMV6-A-GFP, pCMV6-A-GFP-MEG3, respectively (nuclear and plasmic protein). LMNB1 (nuclear protein) or β-actin as an internal control. **E** Immunofluoresence staining (IFS) with anti-PKM2 in liver cancer cells Hep3B cell lines transfected with pCMV6-A-GFP, pCMV6-A-GFP-MEG3, respectively; scale bars,100 μm

**Fig. 6:**
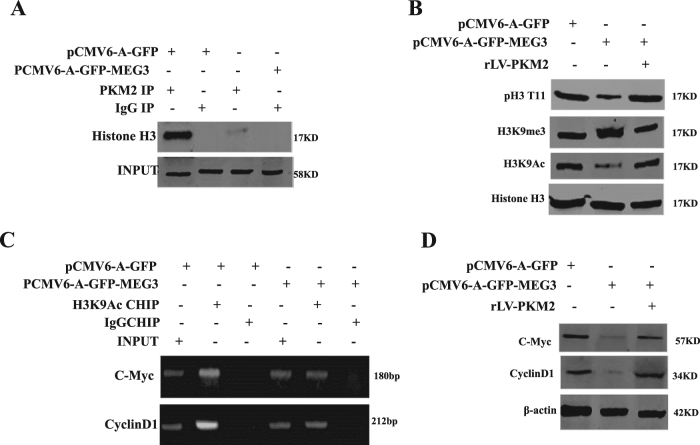
MEG3 inhibits C-myc and CyclinD1 dependent on PKM2. **A** Co-immunoprecipitation (IP) with anti-PKM2 followed by Western blotting with Histone H3 in the Hep3B cell lines transfected with pCMV6-A-GFP, pCMV6-A-GFP-MEG3, respectively. IgG IP as negative control. INPUT refers to Western blotting with Histone H3. **B** Western blotting analysis with anti-pH3T11, anti- H3K9me3, anti-H3K9Ac in liver cancer cells Hep3B cell lines transfected with pCMV6-A-GFP, pCMV6-A-GFP-MEG3, pCMV6-A-GFP-MEG3 plus pcDNA3-PKM2, respectively. β-actin as an internal control. **C** Chromatin immunoprecipitation (CHIP) with anti-H3K9Ac followed by PCR with C-Myc promoter and CyclinD1 promoter primers in liver cancer cells Hep3B cell lines transfected with pCMV6-A-GFP, pCMV6-A-GFP-MEG3, respectively. IgG CHIP as negative control. C-Myc promoter and CyclinD1 promoter as INPUT. **D** Western blotting analysis with anti-C-myc, anti- cyclinD1 in liver cancer cells Hep3B cell lines transfected with pCMV6-A-GFP, pCMV6-A-GFP-MEG3, pCMV6-A-GFP-MEG3 plus pcDNA3-PKM2, respectively. β-actin as an internal control

To explore whether MEG3 could influence the function of PKM2, we selected PKM2-upregulated C-Myc and CyclinD1. At first, the results showed that PKM2 3′-UTR luciferase activity was significantly decreased in pCMV6-A-GFP-MEG3 group compared to the control group (*P* < 0.01) (Fig. 4d). Furthermore, MEG3 could not significantly alter the loading of CTCF and RNA polII on the promoter region of PKM2 (Fig. 4e). Thus, the PKM2 mRNA was significantly unchanged in pCMV6-A-GFP-MEG3 group compared to the control group (Fig. 4f). Significantly, the PKM2 expression was significantly decreased in pCMV6-A-GFP-MEG3 group compared to the control group (Fig. 4g). However, when the miR122 was inhibited, the PKM2 expression was significantly unaltered in pCMV6-A-GFP-MEG3 group compared to the control group (Fig. 4h). Moreover, MEG3 could reduce the ERK1/2 expression (Fig. 5a) and the interplay between ERK1/2 and pPKM2(Ile 429/Leu 431) (Fig. 5b). Therefore, The PKM2(ser37) expression was significantly decreased in pCMV6-A-GFP-MEG3 group compared to the control group (Fig. 5c). Furthermore, the nuclear PKM2 was significantly reduced in pCMV6-A-GFP-MEG3 group compared to the control group (Fig. 5d, e). Strikingly, the interaction between PKM2 and Histone H3 was significantly decreased in pCMV6-A-GFP-MEG3 group compared to the control group (Fig. 6a). Thus, MEG3 decreased pHiatone H3(T11), H3K9Ac, and increased H3K9me3. However, PKM2 knockdown abrogated the MEG3 action (Fig. 6b). Furthermore, MEG3 decreased the loading of H3K9Ac on CyclinD1 and C-Myc promoter region (Fig. 6c). Ultimately MEG3 decreased the expression of CyclinD1 and C-Myc. However, the expression of CyclinD1 and C-Myc did not alter in Hep3B cell line with MEG3 overexpression plus PKM2 knockdown (Fig. 6d). Taken together, these observations suggest that MEG3 decreased the PKM2 expression and nuclear location dependent on miR122, and then inhibited CyclinD1 and C-Myc via PKM2.

### MEG3 promotes β-catenin degradation through ubiquitin–proteasome system dependent on PTEN

**Fig. 7:**
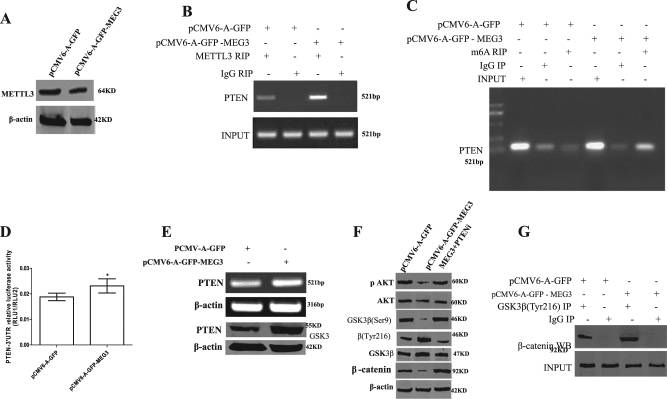
MEG3 inhibits the interaction between GSK3β(Try216) and β-catenin. **A** Western blotting analysis with anti-METTL3 in liver cancer cells Hep3B cell lines transfected with pCMV6-A-GFP, pCMV6-A-GFP-MEG3, respectively. β-actin as an internal control. **B** RNA immunoprecipitation (RIP) with anti-METTL3 followed by RT-PCR with PTEN primers. IgG RIP as negative control. RT-PCR for PTEN as INPUT. **C** RNA immunoprecipitation (RIP) with anti-m6A followed by RT-PCR with PTEN primers. IgG RIP as negative control. RT-PCR for PTEN as INPUT. **D** PTEN 3′-UTR luciferase activity assay. Data are means of values from three independent experiments, mean ± SEM. **, *P* < 0.01; *, *P* < 0.05. **E** Western blotting analysis with anti-PTEN and RT-PCR with PTEN primers.** F** Western blotting analysis with anti-AKT, anti-pAKT, anti-GSK3β(ser9), anti-GSK3β(Tyr216), anti-GSK3β, and anti-β-catenin in liver cancer cells Hep3B cell lines. **G** Co-immunoprecipitation (IP) with anti-GSK3β(Tyr216) followed by Western blotting with anti-β-cateninin. IgG IP as negative control. INPUT refers to Western blotting with anti-β-catenin

**Fig. 8:**
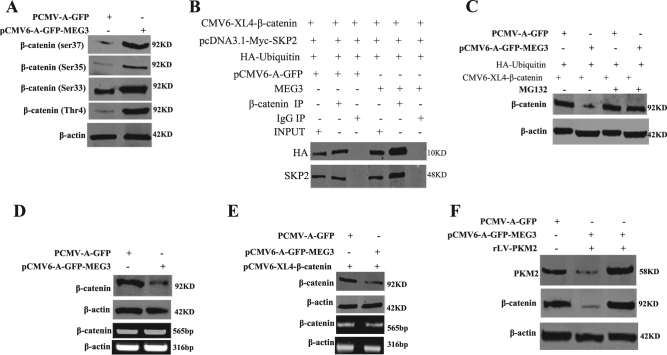
MEG3 promotes β-catenin degradation through ubiquitin–proteasome system. **A** Western blotting analysis with anti-β-catenin(ser33), anti-β-catenin(ser35), anti-β-catenin(ser37), anti-β-catenin(Thr4). β-actin as an internal control. **B** Co-immunoprecipitation (IP) with anti-β-catenin followed by Western blotting with anti-HA and anti-Skp2. IgG IP as negative control. **C** Hep3B Cells were treated  with MG132(50μM) for 6 h at proper after transfection according to the manufacturer’s instructions. Western blotting analysis with anti-β-catenin in liver cancer cells. **D** Western blotting analysis with β-catenin and RT-PCR with β-catenin primers in liver cancer cells Hep3B cell lines transfected with pCMV6-A-GFP, pCMV6-A-GFP-MEG3, respectively. β-actin as an internal control. **E** Western blotting analysis with anti-β-catenin and RT-PCR with β-catenin primers in liver cancer cells Hep3B cell lines transfected with pCMV6-A-GFP plus pCMV6-XL4-β-catenin(FL), pCMV6-A-GFP-MEG3 plus pCMV6-XL4-β-catenin(FL), respectively. β-actin as an internal control. **F** Western blotting analysis with anti-β-catenin in liver cancer cells Hep3B cell lines transfected with pCMV6-A-GFP, pCMV6-A-GFP-MEG3, and pCMV6-A-GFP-MEG3 plus rLV-PKM2 (infected), respectively. β-actin as an internal control

Given MEG3 could inhibit hepatocarcinogenesis, we consider to address whether MEG3 could regulate oncogenes, e.g., β-catenin. At first, we investigated whether MEG3 could alter the expression of PTEN. Although MEG3 did not influence the expression of METTL3 (a enzyme of mRNA methylation) (Fig. 7a), however, MEG3 overexpression increased the interaction between METTL3 and PTEN 3-UTR (Fig. 7b). Therefore, MEG3 overexpression increased the PTEN 3-UTR mRNA methylation compared to control (Fig. 7c). Furthermore, MEG3 overexpression increased the PTEN 3-UTR luciferase activity compared to control (*P* < 0.01) (Fig. 7d). The expression of PTEN was significantly increased on the transcriptioanal and translational level in MEG3 overexpressing group compared to control (Fig. 7e). Therefore, excessive MEG3 overexpression decreased the expression of pAKT, GSK3β(Ser9), β-catenin, and increased the expression of GSK3β(Tyr216). However, PTEN overexpression abrogated the MEG3 action (Fig. 7f). Interestingly, MEG3 overexpression increased the interaction between GSK3β(Tyr216) and β-catenin (Fig. 7g). Thus, excessive MEG3 increased the expression and modification of β-catenin (ser33, ser35, ser37, and Thr4) (Fig. 8a). Furthermore, MEG3 overexpression increased the interaction between β-catenin and HA-Ub, Skp2 (Fig. 8b). Strikingly, MEG3 overexpression increased the degradation of β-catenin. In particular, MEG132 abrogated the MEG3 action (Fig. 8c). Ultimately, excessive MEG3 decreased the expression of β-catenin. However, MEG3 overexpression could not alter the transcription of β-catenin (Fig. 8d, e). Excessive PKM2 could rescue MEG3-mediated β-catenin degradation (Fig. 8f). Taken together, these observations suggest that MEG3 increases the degradation of β-catenin through ubiquitin–proteasome system dependent on PTEN.

### MEG3 inhibits β-catenin activity through PKM2 reduction and PTEN increase

**Fig. 9:**
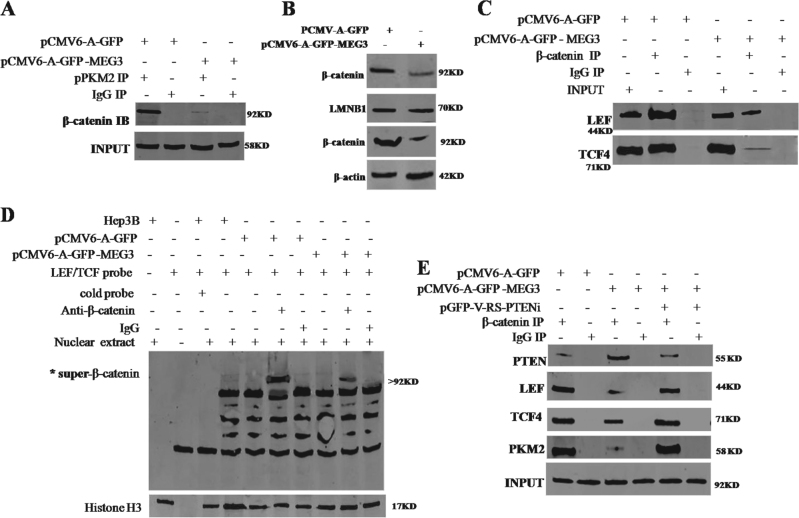
MEG3 inhibits the interaction among β-catenin LEF, TCF4 through PKM2 reduction and PTEN increase. **A** Co-immunoprecipitation (IP) with anti-pPKM2 followed by Western blotting with anti-β-catenin in the Hep3B cell lines transfected with pCMV6-A-GFP, pCMV6-A-GFP-MEG3, respectively. IgG IP as negative control. INPUT refers to Western blotting with anti-β-catenin. **B** Western blotting analysis with anti-β-catenin in liver cancer cells Hep3B cell lines transfected with pCMV6-A-GFP, pCMV6-A-GFP-MEG3, respectively (nuclear and plasmic protein). LMNB1 (nuclear protein) or β-actin as an internal control. **C** Co-immunoprecipitation (IP) with anti-β-catenin followed by Western blotting with anti-LEF and anti-TCF4 in the Hep3B cell lines transfected with pCMV6-A-GFP, pCMV6-A-GFP-MEG3, respectively. IgG IP as negative control. INPUT refers to Western blotting with anti-LEF and anti-TCF4. **D**. Super-EMSA (gel-shift) with biotin-LEF/TCF 4 probe and anti-β-catenin antibody. The intensity of the band was examined by Western blotting with anti-biotin. Histone H3 as an internal control. **E**. Co-immunoprecipitation (IP) with anti-β-catenin followed by Western blotting with anti-PTEN, anti-LEF, anti-TCF4, anti-PKM2 in the Hep3B cell lines transfected with pCMV6-A-GFP, pCMV6-A-GFP-MEG3, pCMV6-A-GFP-MEG3 plus pGFP-V-RS-PTEN, respectively. IgG IP as negative control. INPUT refers to Western blotting with anti-PTEN, anti-LEF, anti-TCF4, anti-PKM2

**Fig. 10:**
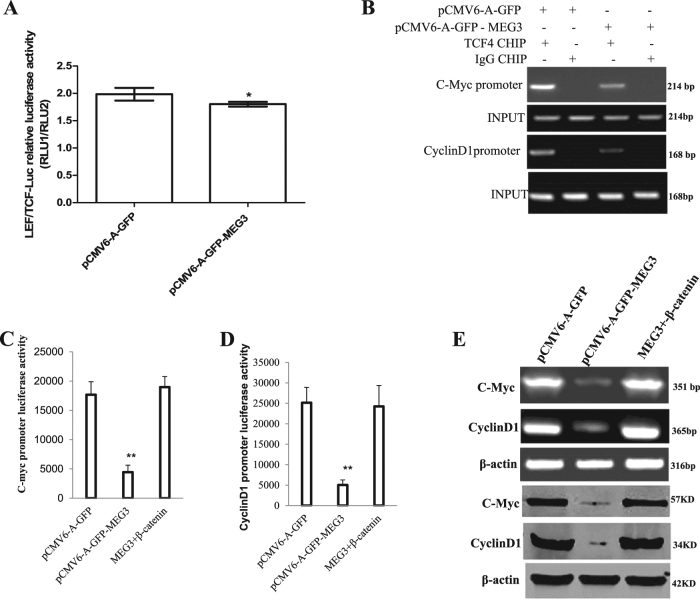
MEG3 inhibits the exptrssion of C-myc and CyclinD1 through β-catenin. **A** LEF/TCF4 luciferase activity assay in liver cancer cells Hep3B cell lines transfected with pCMV6-A-GFP, pCMV6-A-GFP-MEG3, respectively. Data are means of values from three independent experiments, mean ± SEM. **, *P* < 0.01; *, *P* < 0.05. **B** Chromatin immunoprecipitation (CHIP) with anti-TCF4 followed by PCR with C-Myc promoter and CyclinD1 promoter primers in liver cancer cells Hep3B cell lines transfected with pCMV6-A-GFP, pCMV6-A-GFP-MEG3, respectively. IgG CHIP as negative control. C-Myc promoter and CyclinD1 promoter as INPUT. **C** C-myc promoter luciferase activity assay in liver cancer cells Hep3B. **D** CyclinD1 promoter luciferase activity assay in liver cancer cells Hep3B. **E** RT-PCR and Western blotting analysis of C-myc and CyclinD1 in liver cancer cells Hep3B cell lines transfected with pCMV6-A-GFP, pCMV6-A-GFP-MEG3, pCMV6-A-GFP-MEG3 plus pCMV6-XL5-β-catenin, respectively. β-actin as an internal control

To explore whether MEG3 influenced β-catenin activity, we analyzed the activity of β-catenin co-activators LEF and TCF-4 in liver cancer cells. As shown in Fig. 9a, MEG3 overexpression decreased the interaction between pPKM2 and β-catenin. Thereby, MEG3 overexpression decreased the expression and nuclear localization of β-catenin (Fig. 9b). Furthermore, MEG3 overexpression decreased the interaction between β-catenin and LEF, TCF4 in liver cancer cells. (Fig. 9c). Therefore, MEG3 overexpression decreased the binding of β-catenin to LEF/TCF4 probe (Fig. 9d). In particular, MEG3 overexpression increased the interplay between β-catenin and PTEN, and decreased the interplay between β-catenin and LEF, TCF4. However, the MEG3 action was abrogated when the PTEN was knocked down (Fig. 9e). MEG3 overexpression decreased the activity of LEF/TCF4 (Fig. 10a). Furthermore, MEG3 overexpression decreased the loading of C-myc promoter region and CyclinD1 promoter region (Fig. 10b). Thereby, MEG3 overexpression decreased the promoter luciferase of C-myc (Fig. 10c) and CyclinD1 (Fig. 10d). However, the MEG3 action was abrogated when the β-catenin was knocked down (Fig. 10c, d). Finally, MEG3 overexpression decreased the expression of C-myc and CyclinD1 on the level of transcription and translation. However, the MEG3 action was abrogated when β-catenin was knocked down (Fig. 10e). Collectively, these observations suggest that MEG3 inhibits β-catenin activity through PKM2 reduction and PTEN increase in liver cancer cells.

### β-catenin determines the functions of MEG3 suppressor

**Fig. 11:**
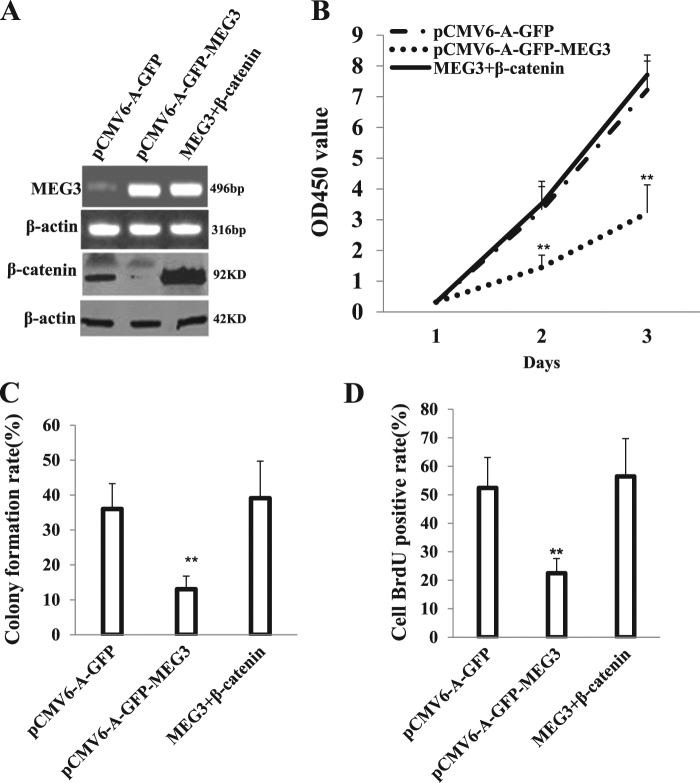
Rescued β-catenin abrogated the MEG3 suppressor function in vitro. **A** Western blotting analysis with anti-β-catenin and PCR-PCR with MEG3 primers in the Hep3B cell lines transfected with pCMV6-A-GFP, pCMV6-A-GFP-MEG3, pCMV6-A-GFP-MEG3 plus pCMV6-XL5-β-catenin, respectively. β-actin served as an internal control. **B** Cells growth assay using CCK8. Each value was presented as mean ± standard error of the mean (SEM). **, *P* < 0.01. **C** Cell BrdU staining assay. Each value was presented as mean ± standard error of the mean (SEM). **, *P* < 0.01. **D** Cells soft agar colony formation assay. Each value was presented as mean ± standard error of the mean (SEM). **, *P* < 0.01

**Fig. 12:**
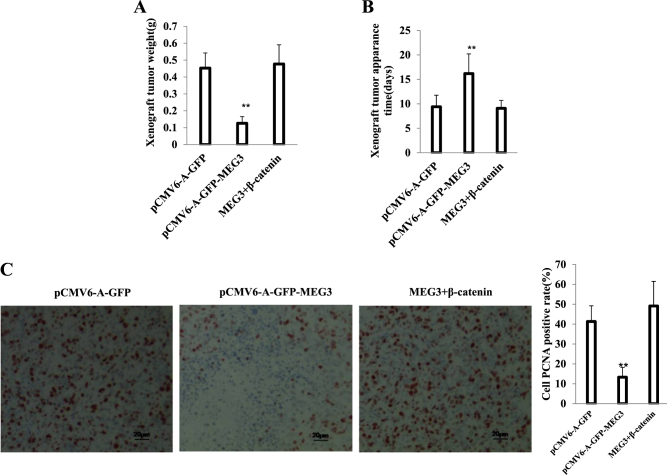
β-catenin determines MEG3 suppressor function in vivo. **A** Tumorigenesis test in vivo. The mice were stratified and the tumors were recovered. The wet weight of each tumor was determined for each mouse. Each value was presented as mean ± standard error of the mean (SEM). **, *P* < 0.01. **B** The appearance time of each tumor was determined for each mouse. Each value was presented as mean ± standard error of the mean (SEM). **, *P* < 0.01. **C** A portion of each tumor was fixed in 4% paraformaldehyde and embedded in paraffin for histological hematoxylin–eosin (HE) staining and PCNA staining (DAB stainning, original magnification ×100).

To explore whether MEG3 suppressor function is associated with β-catenin, we established three stable Hep3B cell lines (pCMV6-A-GFP, pCMV6-A-GFP-MEG3, pCMV6-A-GFP-MEG3 plus pCMV6-XL4-β-catenin). As shown in Fig. 11a, compared to control, MEG3 was overexpressed in stable Hep3B cell lines transfected with pCMV6-A-GFP-MEG3, pCMV6-A-GFP-MEG3 plus pCMV6-XL4-β-catenin, and β-catenin was overexpressed in stable Hep3B cell lines transfected with pCMV6-A-GFP-MEG3 plus pCMV6-XL4-β-catenin and decreased in stable Hep3B cell lines transfected with pCMV6-A-GFP-MEG3. Next, we detected the cell proliferation. As shown in Fig. 11b, excessive MEG3 significantly decreased the growth of liver cancer cell Hep3B compared to the control cells (*P* < 0.01). However, HULC plus β-catenin did not significantly alter the growth of liver cancer cells (*P* > 0.05). Moreover, MEG3 overexpression significantly decreased the BrdU positive rate compared to the control cells (22.46 ± 5.18% versus 52.4 ± 10.71%, *P* = 0.007949 <0.01). However, MEG3 plus β-catenin did not significantly alter the BrdU positive rate of liver cancer cells (56.43 ± 13.27% versus 52.4 ± 10.71%, *P* = 0.3793616 >0.05) (Fig. 11c). We further performed colony formation assay and observed a significant decrease in colony formation efficiency rate in excessive MEG3 (13.04 ± 3.76% versus 36.02 ± 7.26%, *P* = 0.004634 <0.01). However, MEG3 plus β-catenin did not significantly alter the colony formation rate of liver cancer cells (39.12 ± 10.6% versus 36.02 ± 7.26%, *P* = 0.1985 >0.05) (Fig. 11d). Furthermore, the three stable Hep3B cell lines were injected subcutaneously into athymic Balb/C mice. As shown in Fig. 12a, when MEG3 was overexpressed, the average xenograft tumor weight decreased approximately 0.27 folds compared to the corresponding control group (0.1267 ± 0. 039 g versus 0.4533 ± 0.089 g, *n* = 6, *P* = 0.000347 <0.01). However, MEG3 plus β-catenin did not significantly alter the xenograft tumor weight (0.4767 ± 0.1138 g versus 0.4533 ± 0.089 g, *n* = 6, *P* = 0.251967 >0.01). On the other hand, when MEG3 was overexpressed, the average xenograft tumor appearance time was significantly increased compared to the corresponding control group (16.18 ± 4.02 days versus 9.4 ± 2.37 days, *n* = 6, *P* = 0.003787 <0.01). However, MEG3 plus β-catenin did not significantly alter the xenograft tumor appearance time (9.08 ± 1.62 days versus 9.4 ± 2.37 days, *n* = 6, *P* = 0.41429 >0.05) (Fig. 12b). Moreover, the proliferation index (calculated as percentage of PCNA-positive cells) was significantly lower in MEG3 overexpressing xenograft tumors compared to the control group (13.31 ± 4.91% versus 41.33 ± 7.88%, *P* = 0.02939 <0.05). However, MEG3 plus β-catenin did not significantly alter the PCNA positive rate of liver cancer cells (49.13 ± 12.38% versus 41.33 ± 7.88%, *P* = 0.237289 >0.05) (Fig. 12c). Compared to the control, the expression of β-catenin and PKM2 is significant in the MEG3 overexpressing group (Fig. 12d). This suggested that MEG3 inhibited cell growth, colony formation ability, and cell growth in vivo. However, β-catenin overexpression abrogated the MEG3 action. Taken together, β-catenin determines the MEG3 suppressor function in liver cancer cells.

## Discussion

**Fig. 13:**
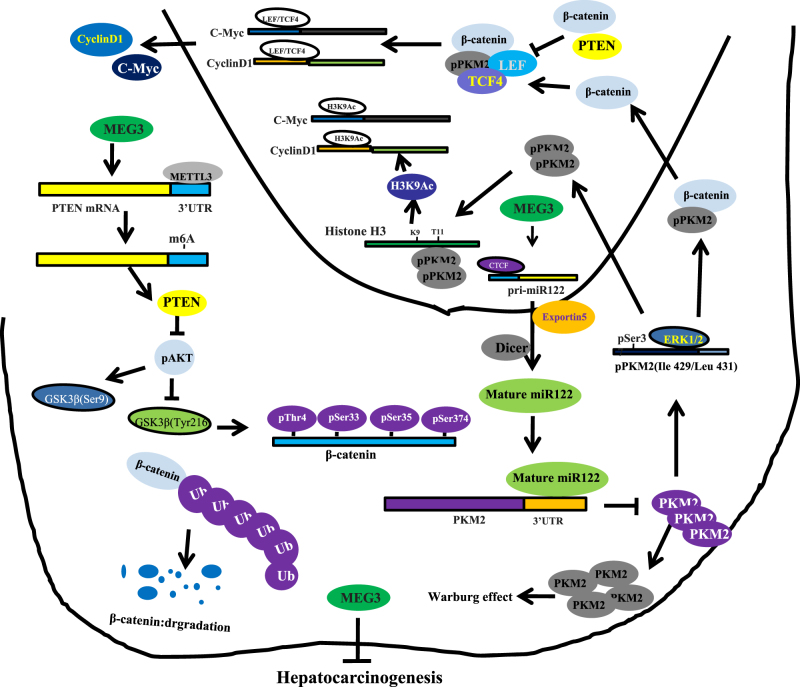
The schematic illustrates a model that long noncoding RNA MEG3 suppresses liver cancer cells growth through β-catenin by activating PKM2 and inactiviating PTEN. MEG3 promotes the expression and maturation of miR122 which targets PKM2. Therefore, MEG3 decreased PKM2 expression and nuclear location dependent on miR122. Furthermore, MEG3 inhibited CyclinD1 and C-Myc via PKM2 in liver cancer cells. Strikingly, MEG3 promotes β-catenin degradation through ubiquitin–proteasome system dependent on PTEN. Moreover, MEG3 inhibits β-catenin activity through PKM2 reduction and PTEN increase. Furthermore, we found that excessive β-catenin rescued the effect of MEG3 in liver cancer

It has been confirmed that MEG3 encodes an lncRNA which is suggested to function as a tumor suppressor and has been shown to involve in a variety of cancers. Our studies are now indicated to evaluate the effects of MEG3 in liver cancer cells. Our findings demonstrate that MEG3 inhibits the malignant progression of liver cancer cells in vitro and in vivo. Mechanistically, MEG3 promotes the expression and maturation of miR122 which targets PKM2. Therefore, MEG3 decreased the PKM2 expression and nuclear location dependent on miR122. Furthermore, MEG3 inhibited CyclinD1 and C-Myc via PKM2 in liver cancer cells. Strikingly, MEG3 promotes β-catenin degradation through ubiquitin–proteasome system dependent on PTEN. Moreover, MEG3 inhibits β-catenin activity through PKM2 reduction and PTEN increase. Furthermore, we found that excessive β-catenin rescued the effect of MEG3 in liver cancer (Fig. 13). To our knowledge, this is the first report demonstrating that lncRNA MEG3 suppresses liver cancer cells growth through β-catenin by activating PKM2 and PTEN.

To date, accumulating evidence indicates that MEG3 plays a critical role in cancer progression and metastasis. Previous studies suggested that MEG3 functioned through the activation of p53; however, the functional properties of MEG3 remain obscure and their relevance to human diseases is under continuous investigation^43^. Crosstalk between MEG3 and miR-1297 regulates the growth of testicular germ cell tumor through PTEN/PI3K/AKT pathway^44^. MEG3 may be an underlying therapeutic target for LUAD functioning as ceRNAs for the regulation of miRNA-mRNA in lung adenocarcinoma^45^. In addition, MEG3 was decreased in primary endometrial stromal cells (ESCs) in response to TGF-β1 stimulation^46^.

Evidently, our results indicate that the involvement of MEG3 inhibition of liver cancer cell growth is supported by results from two parallel sets of experiments:^1^ MEG3 is downregulated and is postively associated with miR122, PTEN and negatively associated with PKM2, β-catenin expression in human liver cancer tissue^2^. MEG3 inhibits malignant progression of liver cancer cells in vitro and in vivo. Our observations demonstrated that MEG3 is crucial for the inhibition of cell growth and viability in liver cancer cells. According to the aforementioned findings, MEG3 is a tumor suppressor.

Of significance, our findings clearly showed that MEG3 promotes the expression and maturation of miR122 which targets PKM2 and inhibits the expression of PKM2. Studies indicate that alcoholic hepatitis accelerates early hepatobiliary cancer by increasing stemness and miR122-mediated HIF-1α activation^47^. Furthermore, miR122 is implicated as a regulator of physiological and pathophysiological processes in the liver. Gα12 overexpressed in hepatocellular carcinoma reduces microRNA-122 expression via HNF4α inactivation, which causes c-Met induction^48^. A quantitative mathematical model of HCV-induced miR-122 sequestration proposes that such miR122 inhibition by HCV RNA may result in global de-repression of host miR-122 targets, providing an environment fertile for the long-term oncogenic potential of HCV^49^.

Accordingly, reduction in PKM2 may partly contribute to MEG3-mediated inhibition of liver cancer cell growth. Our findings in this study provide a novel evidence for an active role of PKM2 in MEG3-mediated inhibition of liver cancer cell growth. This assertion is based on several observations^1^: MEG3 decreased the PKM2 expression dependent on miR122^2^. MEG3 inhibits nuclear localization and function of PKM2 dependent on miR122^3^. MEG3 inhibits the expression of cyclinD1 and C-Myc via PKM2. These findings are noteworthy, given that PKM2 is and functions as a key oncogene to mediate various biological processes including cell proliferation and differentiation. Moreover, PKM2 is associated with cancer differention^50,51^. Pyruvate kinase M2 activates mTORC1 by phosphorylating AKT1S1^52^ and PKM2 promotes tumor angiogenesis by regulating HIF-1α through NF-κB activation^53^. In particular, cytosolic PKM2 stabilizes mutant EGFR protein expression through regulating HSP90-EGFR association^54^. PKM2 promotes stemness of breast cancer cell by through Wnt/β-catenin pathway^55^. In addition, miR675 upregulates lncRNA H19 through activation of EGR1 in human liver cancer^56^. However, a study failed to observe PKM2-dependent transfer of phosphate from ATP directly to protein^57^. Furthermore, our findings indicated that MEG3 inhibited the expression of C-myc, whereas C-myc decides the reprogramming metabolism of cancer^58^.

Strikingly, we also demonstrated that MEG3 is closely associated with PTEN and β-catenin in liver cancer cells. This assertion is based on several observations^1^: MEG3 increased the expression and phosphorylation of PTEN^2^. MEG3 promotes β-catenin degradation through ubiquitin–proteasome system dependent on PTEN^3^. MEG3 inhibits β-catenin activity through PKM2 reduction and PTEN increase in liver cancer cells^4^. MEG3 inhibited cell growth, colony formation ability, and cell growth in vivo. However, β-catenin overexpression abrogated the MEG3 action. β-catenin determines MEG3 suppressor function in liver cancer cells.

It is well known that PTEN is a lipid phosphatase that converts phosphatidylinositol 3,4,5-phosphate (PIP3) to phosphatidylinositol 4,5-phosphate (PIP2) and plays a critical role in the regulation of tumor growth^59,60^. Notch promotes tumor metastasis in a prostate-specific PTEN-null mouse model^61^. miR-18a promotes cell proliferation of esophageal squamous cell carcinoma cells by increasing cylin D1 via regulating PTEN-PI3K-AKT-mTOR signaling axis^62^. Furthermore, PTEN is a key molecular controller of the PI3K signaling, and PI3K-PTEN dysregulation leads to mTOR-driven upregulation of the core clock gene BMAL1 in normal and malignant epithelial cells^63^. In particular, PTEN negatively regulates mTORC2 formation and signaling in grade IV glioma via Rictor hyperphosphorylation at Thr1135 and directs the mode of action of an mTORC1/2 inhibitor^64^. In addition, SOX7 co-regulates Wnt/β-catenin signaling with Axin-2^65^ and FAK promotes osteoblast progenitor cell proliferation and differentiation by enhancing Wnt signaling^66^. Moreover, FOXKs promote Wnt/β-catenin signaling by translocating DVL into the nucleus^67^ and ECM1 regulates tumor metastasis and CSC-like property through stabilization of β-catenin^68^. A report shows that microRNA-153 promotes β-catenin activation in hepatocellular carcinoma through the suppression of WWOX^69^. Also, there is a regulation function between Wnt/β-catenin signaling and PI3K/Akt survival pathway^70^.

## Conclusion

The present study depicts a novel evidence for MEG3 that plays inhibiting tumorigenesis roles by downregulating PKM2 and β-catenin in liver cancer cells, which may have potential therapeutic significance. Alteration of the expression of lncRNAs MEG3 may also mediate changes at an epigenetic level to affect gene expression and contribute to inhibiting hepatocarcinogenesis. MEG3 overexpression in combination with blocking PKM2 and β-catenin might represent a promising treatment strategy targeting tumors. Our study for the first time demonstrated that MEG3 acts as a tumor suppressor by negatively regulating the activity of PKM2 and β-catenin in hepatocarcinogenesis and might serve as a prognostic biomarker and molecular therapeutic target.
